# Impact of a culinary medicine intervention on diet and health metrics in patients with type 2 diabetes and elevated body mass index

**DOI:** 10.1371/journal.pone.0347040

**Published:** 2026-05-20

**Authors:** Natalia I. Heredia, Logan R. Thornton, Diana C. Guevara, Natalia Williams, Grace Marcelo-Ramirez, Joanne Chow, Lorna H. McNeill, Karen Basen-Engquist, Ruth Rechis, Deanna M. Hoelscher

**Affiliations:** 1 UTHealth Houston School of Public Health, Houston, Texas, United States of America; 2 Healthcare Transformation Initiatives, UTHealth Houston John P. and Kathrine G. McGovern Medical School, Houston, Texas, United States of America; 3 The University of Texas MD Anderson Cancer Center, Houston, Texas, United States of America; 4 Michael and Susan Dell Center for Health Living, UTHealth School of Public Health, Austin, Texas, United States of America; University of Milan, ITALY

## Abstract

**Purpose:**

Culinary Medicine (CM), an educational approach blending the art of cooking with the science of medicine, has shown promise in improving diet and health outcomes. We conducted a mixed-methods, quasi-experimental evaluation of a CM program to promote cooking and healthy dietary intake.

**Methods:**

Patients with type 2 diabetes and elevated body mass index (>25) were recruited from primary care clinics in low-income neighborhoods. The program, delivered by a dietitian, includes five weekly 90-minute sessions on basic cooking skills with behaviorally-based nutrition education. An explanatory sequential mixed-methods pilot study with a pre-post survey design and post-program interviews was used to assess improvements in dietary behaviors, nutrition knowledge, and cooking skills.

**Results:**

A total of 89 individuals enrolled (58 intervention, 31 control). Fifty (86%) intervention participants attended the sessions, and 45 (78%) completed post-intervention surveys, while 22 (71%) control participants completed post-intervention surveys. Compared to control, the intervention group showed statistically significant improvements in perceived health (Adjusted Odds Ratio = 16.15, 95% CI 3.47,75.23, p < 0.001) and cooking self-efficacy (Adjusted *β* ***=*** 0.43, 95% CI 0.03,0.84, p = 0.04), and a decrease in perceived barriers to healthy eating (Adjusted β = *−*0.64, 95% CI −1.21,-0.08, p = 0.03). We found a statistically significant decrease in HbA1c in the intervention group from pre- to post-test, though between-group comparisons were not statistically significant. All other behavioral, psychosocial, and biometric variables were non-significant but trended in the expected direction.

**Conclusions:**

This study is feasible and acceptable. The CM program effectively promotes cooking skills and healthy eating among low-income patients with type 2 diabetes and elevated body mass index.

## Introduction

Diabetes and obesity are especially prevalent cardiometabolic diseases that are leading causes of illness, death, and healthcare spending in the United States (U.S.) [1]. Moreover, the prevalence of both conditions has continued to increase in the U.S. [2–4]. These conditions often co-occur and share similar health risk behaviors (unhealthy diet, sedentary lifestyle, etc.), and they pose enormous direct and indirect costs [1]. A healthy diet is key to managing both conditions [5,6], but diet quality is generally low in the U.S. [7,8]. Moreover, many patients with diabetes lack the knowledge and skills needed to be able to consistently eat healthier. One way to sustain healthy eating is to cook meals at home [9–12]; however, cooking skills in the U.S. have decreased over time [13]. Lack of knowledge and skills to select healthy foods and cook healthy meals is considered a barrier to dietary changes [14,15]. Therefore, there is an urgent need to equip patients with cooking skills and nutrition knowledge.

Even with the increasing use of Glucagon-like peptide 1 receptor agonists (GLP-1s) and combination medications for individuals with type 2 diabetes and/or elevated Body Mass Index (BMI) [16], there are a myriad of reasons for the continued need for interventions that support changes in dietary behavior [17]. While GLP-1s are effective, they remain expensive and inaccessible to many communities and have gastrointestinal side effects that need to be carefully managed; further, their discontinuation without sustained lifestyle changes often leads to weight regain [17–19]. Therefore, there are continued calls to combine dietary lifestyle interventions with the use of GLP-1s [17].

We developed a culturally inclusive culinary medicine and nutrition education program, Nourishing the Community Through Culinary Medicine (NCCM). This program provides a combination of nutrition education, cooking instruction, and financial support to purchase ingredients for each class. While this intervention was previously tested in an uncontrolled trial [20], the present work uses a pilot non-randomized trial design. The purpose of this study is to assess the feasibility and acceptability of the NCCM intervention, as well as describe its effectiveness on anthropometric, clinical, behavioral and psychosocial variables, as compared to a control group, in patients living with type 2 diabetes and elevated BMI (>25).

## Materials and methods

### Study design

This pilot study was a unblinded quasi-experimental design, with designated (non-randomized) intervention and control sites from which we recruited patients. This study was reviewed and approved by the UTHealth Houston Institutional Review Board and registered at clinicaltrials.gov (NCT06096506).

### Intervention

The NCCM intervention has been described previously [21]. Briefly, five 90-minute, weekly classes were live-streamed via a video conferencing platform from the study site teaching kitchen, with participants cooking and engaging virtually from their home kitchens. Each week, the dietitian leading the course walked participants through a recipe designed to teach fundamental cooking skills (e.g., knife skills, sautéing, steaming). While foods were simmering or in the oven, the dietitian showed educational videos conveying nutrition information and facilitated discussions. The curriculum is based on Social Cognitive Theory [22,23], especially focusing on the constructs of outcome expectations, self-efficacy, behavioral capability (knowledge and skills), and social support. The recipes used during the sessions were developed with the intention of being culturally inclusive, with recipes pulling from an array of cultural traditions, paired with a discussion on how to personalize any future uses of the recipes for individual, family or cultural preferences. Participants were provided a $40 grocery card and shopping list prior to each class (a total of $200 across five classes) to purchase ingredients. We offered classes on weekday evenings, and the recipes cooked during class provided enough food for a family of four. Additionally, asynchronous virtual educational content (cooking skills videos, animated nutrition education videos, and additional recipes) was provided to engage participants beyond the synchronous sessions [24]. Prior to starting the study and intervention, we presented to and consulted with the Be Well^TM^ Acres Homes Steering Committee to seek input.

### Study population

From January to August 2024, we recruited and screened patients from a clinic system serving the greater Houston area. The intervention clinics served a persistent poverty neighborhood (≥20% of an area’s population living in poverty for 30 or more years), while the control clinics served a demographically similar patient population in two different Houston neighborhoods. Patients were included if they were adults (18–70 years of age) receiving care at a participating clinic, had a diagnosis of type 2 diabetes, had an elevated BMI (≥25), had HbA1c labs and clinic-assessed weight completed within the last 3 months, spoke English or Spanish, and were able to obtain groceries before each class. We excluded patients without the technological equipment needed to participate (e.g., reliable internet and device – cell phone, tablet or laptop), patients who indicated they could not make the class times and patients with an uncontrolled impairment that interfered with their ability to participate.

A care coordinator working across the clinical sites assessed preliminary patient eligibility (age, type 2 diabetes diagnosis, BMI) to participate in the program and shared a flyer with a study interest form with these patients. Patients who were interested completed the form, including their contact information. Study staff then reached out to explain the study, and if participants consented to eligibility screening, they assessed all additional eligibility criteria. If eligible and still interested, study staff sent a link to the online informed consent, and participants completed written informed consent.

### Data collection

Baseline data collection occurred from January to August 2024. Follow-up data collection occurred from February to October 2024. The primary outcomes of this study were feasibility and acceptability, and secondary outcomes were changes in psychosocial, behavioral, and biometric variables. The feasibility of recruitment and retention was assessed with project records, and acceptability was assessed with in-depth interviews.

We collected self-administered questionnaires electronically via REDCap at baseline and post-intervention follow-up. Participants received a $25 gift card for completing each questionnaire. HbA1c, BMI, and Blood Pressure were collected from usual care visits using Electronic Medical Record (EMR) data. Baseline EMR data included measures within 90 days of starting the program, and post-intervention EMR data included data measured within 90 days of program completion. We also extracted medication data, including anti-hypertensive and diabetes-related medications. Using the medication data available in the EMR over time, we assessed an increase in the type or dose of medications from baseline to post-intervention.

A subset of participants from the intervention group were invited to take part in interviews about their experience with various aspects of the program (e.g., cooking classes, recipes, recruitment, and engagement), aspects they found favorable or unfavorable, and any barriers to participation they encountered. Participants received an additional $25 gift card for interview participation. The interviews lasted approximately 20–45 minutes, were conducted by trained staff, and were recorded for transcription.

No harms were reported in this study.

### Measures

In addition to the EMR data, we used pre- and post-test questionnaires. Perceived health was measured with one item with a 7-point response scale from “Excellent” to “Very Poor” [25]. Daily servings of vegetables and fruits were each measured with a single item adapted from a previously validated instrument, with a 6-point response scale from “None” to “4+ servings” [26]. Frequency of healthy food consumption behaviors was measured with a 7-item validated measure (e.g., “How often do you typically eat other non-fried vegetables like carrots, broccoli, green beans, or other vegetables?”), with response options on a 5-point scale from “Not at all” to “More than once a day” [27]. We created a score by averaging the items, with higher scores indicating greater frequency of eating healthful foods. We measured eating, cooking and shopping behaviors using 8 items adapted from a previously validated survey (e.g., “How often do you plan meals ahead of time?”) with a 5-point response scale from “Never” to “Always” [27]. We averaged the items, with higher scores indicating healthier behaviors (Cronbach’s alpha = 0.79). Perceived barriers to eating fruits and vegetables were measured with 9 items (e.g., “I don’t eat fruits and vegetables as much as I like because they cost too much) with responses on a 5-point Likert scale from “Strongly disagree” to “Strongly agree”. We averaged the items, with higher scores indicating more barriers (Cronbach’s alpha = 0.85). Lastly, we measured cooking self-efficacy with a 5-item scale adapted from prior work (e.g., “How sure are you that you can prepare fresh or frozen green vegetables (e.g., broccoli, spinach)?”) [28]. Response options were on a 5-point Likert scale from “Not at all sure” to “Extremely sure”. We averaged the items, with higher scores indicating greater cooking self-efficacy (Cronbach’s alpha = 0.82).

Demographic data were collected at baseline only for both groups. These included age, sex, race/ethnicity, language spoken at home, annual household income, highest level of education, employment status, health insurance, participation in food and health assistance programs, food insecurity, housing insecurity and housing problems.

### Analyses

We assessed demographic characteristics of the full sample and compared the intervention group to the control group using t-tests for continuous and either Fisher’s exact test or chi-squared test for categorical variables, as appropriate. We used multilevel mixed-effects regression models to assess the association of changes in each psychosocial, behavioral, and biometric outcome between baseline and post-intervention in the intervention group compared to the control group. To account for repeated measures on each subject, multilevel mixed-effects regression models adjusted for ‘subject’ as a random effect were used to obtain estimations for both survey and biometric outcomes. Group and time were included as fixed effects, and group-by-time interaction terms were tested to assess between-group changes over time. We assessed demographic variables at baseline and adjusted for variables that were statistically significantly different between the intervention and control groups, while *a priori* planning to force in age, race/ethnicity, and sex into all models. We also extracted the medication data, created variables to identify the change in the use of anti-hypertensive and anti-diabetic medications, and tested for their potential confounding effects on biometric variables (i.e., if coefficients changed by 10% or more), respectively. We conducted complete case analyses for all variables and samples are provided in the table next to each variable. All analyses were performed using Stata (version 15.1, StataCorp), and *P* ≤ 0.05 was considered statistically significant.

We used the framework approach to analyze in-depth interviews [29]. Two team members familiarized themselves with the data by reading through the transcript (GM, NH) and identifying potential themes and subthemes. One coder (GM) then coded and charted the data into a matrix, with columns for themes/subthemes and rows for participants. Two team members (GM and NH) discussed, refined (as necessary), and collaboratively interpreted the findings.

## Results

**Fig 1:**
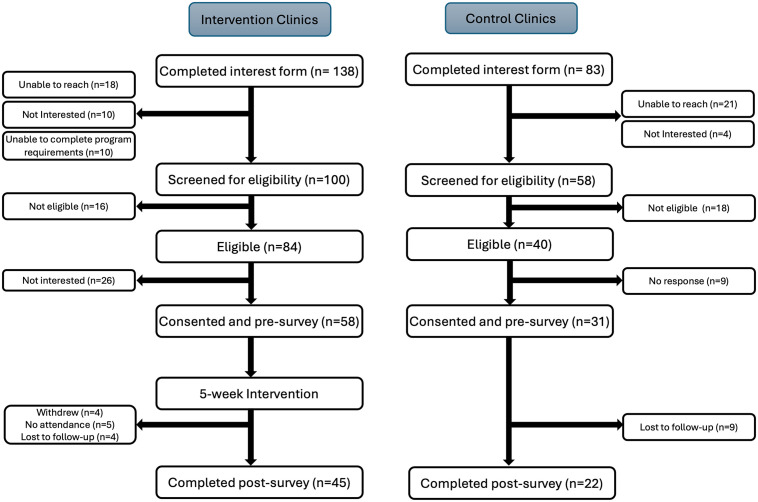
CONSORT diagram of study flow.

The CONSORT diagram is presented in Fig 1; 138 individuals from the intervention clinic sites completed an interest form, of which we reached 120 and then 100 of those were interested in being screened for eligibility. Of those, 84 were eligible and 58 enrolled (69% of eligible). Of the 58 people in the intervention group, 50 participated in at least one intervention session. We had a total of 45 individuals in the intervention group who completed post-intervention data collection (78% of enrolled). For the group recruited from control clinic sites, 83 people completed the interest form, 21 of 83 could not be reached, and 4 of 83 were no longer interested. Of the 58 interested and screened for eligibility, 40 were eligible and 31 ultimately consented and completed the baseline survey (78% of eligible). Of those, 22 completed post-intervention surveys (71% of enrolled). Between the two groups, 89 of 124 eligible individuals enrolled (72%), and 67 of the enrolled individuals (75%) were retained.

The average age of the sample was 55 years, with the control group slightly older (58 years) than the intervention group (53 years; Table 1). The sample was 77% female overall, with a greater percentage of females in the control group (87%) compared to the intervention group (72%). The overall sample was 67% Non-Hispanic Black and 26% Hispanic, though there were statistically significant differences between intervention and control groups, with a greater proportion of Non-Hispanic Black individuals in the intervention group (77%) compared to the control group (46%). Almost half (45%) of the sample had a high school diploma or less.

**Table 1:** Demographic and other sample characteristics.

Characteristics	Total	Intervention Group n = 58	Control Group n = 31	p-value
		mean (SD)	mean (SD)	
**Age**	54.8 (12.0)	53.1(12.6)	58.2 (10.1)	0.061
**# people in the household**	3.0 (1.7)	3.2 (1.8)	2.6(1.3)	0.128
**# children in household** ^a^	0.9 (1.4)	1.1 (1.5)	0.6 (0.9)	0.186
		**n(%)**	**n(%)**	
**Sex**				
Male	20 (22.7)	16 (27.6)	4 (13.3)	0.181
Female	68 (77.3)	42 (72.4)	26 (86.7)	
**Race/ethnicity**				
Hispanic	22 (25.9)	9 (15.8)	13 (46.4)	**0.007**
Non-Hispanic Black	57 (67.1)	44 (77.2)	13 (46.4)	
Non-Hispanic White or Other	6 (7.1)	4 (7.0)	2 (7.2)	
**Language spoken at home**				
English only	70 (78.7)	49 (84.5)	21 (67.7)	0.066
Bilingual	19 (21.3)	9 (15.5)	10 (32.3)	
**Annual household income**				
<=$20,000	43 (52.4)	27 (48.2)	16 (61.5)	0.097
$20,001 to $49,999	21 (25.6)	13 (23.2)	8 (30.8)	
$50,000 or more	18 (22.0)	16 (28.6)	2 (7.7)	
**Highest education level**				
College graduate	15 (18.1)	12 (21.8)	3 (10.7)	0.339
Some College	31 (37.4)	22 (40.0)	9 (32.1)	
High School Graduate	25 (30.1)	15 (27.3)	10 (35.7)	
Without high school degree	12 (14.5)	6 (10.9)	6 (21.5)	
**Employment Status**				
Employed (full time/part time)	19 (22.1)	15 (26.3)	4 (13.8)	0.273
Not employed^b^	67 (77.9)	42 (73.7)	25 (86.2)	
**Health Insurance**				
Yes	69 (78.4)	47 (81.0)	22 (73.3)	0.405
No	19 (21.6)	11 (19.0)	8 (26.7)	
**Participation in health assistance program** ^**c**^				
Yes	63 (70.8)	40 (69.0)	23 (74.2)	0.605
No	26 (29.2)	18 (31.0)	8 (25.8)	
**Participation in food assistance program** ^**d**^				
Yes	42 (47.2)	31 (53.5)	11 (35.5)	0.106
No	47 (52.8)	27 (46.5)	20 (64.5)	
**Food Insecurity**				
Yes	67 (83.8)	42 (84.0)	25 (83.3)	0.938
No	13 (16.3)	8 (16.0)	5 (16.7)	
**Increase in type and/or dose of anti-hypertensive medication(s) from baseline to post-intervention?**				
Yes	12 (15.8)	8 (17.4)	4 (13.3)	0.754
No	64 (84.2)	38 (82.6)	26 (86.7)	
**Increase in type and/or dose diabetes-related medications from baseline to post-intervention?**				
Yes	30 (39.5)	21 (45.7)	9 (30.0)	0.172
No	46 (60.5)	25 (54.4)	21 (70.0)	

Notes: ^a^ Children in the household refers to those less than 18, ^b^ not employed refers to those who are unemployed, homemaker, student, retired, or disabled, ^c^ Medicaid/Texas Health Steps, Medicare, or CHIP etc; ^d^ WIC, SNAP or free/reduced meals at school etc. Table only includes available data (missing data for each variables not included).

In unadjusted analyses, we found a statistically significant increase in perceived health, servings of fruits and vegetables, frequency of healthy food consumption, and cooking self-efficacy, and a decrease in perceived barriers to healthy eating in the intervention group from baseline to post-intervention, as compared to the control group (S1 Table). However, in adjusted analyses that controlled for statistically significantly different variables between the intervention and control groups at baseline (race/ethnicity) and forced in age and sex, the only statistically significant between-group changes remaining were the improvements in perceived health (Adjusted Odds Ratio = 16.15, 95% CI 3.47, 75.23, p < 0.001) and cooking self-efficacy *(Adjusted β* ***=*** 0.43, 95% CI 0.03, 0.84, p = 0.037), and a decrease in perceived barriers to healthy eating *(Adjusted* β = *−*0.64, 95% CI −1.21, −0.08, p = 0.03; Table 2). Servings of fruits and vegetables, frequency of healthy food consumption, and shopping, cooking and eating behaviors also improved more in the intervention group from baseline to post-intervention as compared to the control group, but changes were not statistically significant. In the intervention group alone, all psychosocial and self-reported dietary variables significantly improved in the expected direction between baseline and post-intervention (Table 2). We found a statistically significant decrease in HbA1c pre- to post-test in the intervention group only (Table 3), though the between-group reduction was not statistically significant. We found a marginally significant decrease in systolic blood pressure (SBP) and diastolic blood pressure (DBP) in unadjusted analyses (S2 Table; p = 0.064 and p = 0.086, respectively), though after controlling for race/ethnicity, age, and sex, the relationship was further attenuated and was non-significant (Table 3). However, all biometric variables trended in the expected direction.

**Table 2:** Multivariate adjusted mixed-effects regression assessing the association of the NCCM intervention with psychosocial and behavioral outcomes.

	Intervention Group			Comparison Group			
Variable	Baseline	Post- Intervention	Within Group Changes	Baseline	Post- Intervention	Within Group Changes	Between Group Changes
Ordinal Logistic Regression	*n (%)*		*Adjusted Odds Ratio (95% CI) P-value*	*n (%)*		*Adjusted Odds Ratio (95% CI) P-value*	*Adjusted Odds Ratio (95% CI) P-value*
**Perceived Health**							
Excellent	3 (6.1)	15 (33.3)	**17.13** **(6.24, 47.00)** **p < 0.001**	2 (6.7)	1 (4.8)	1.06 (0.32, 3.50) p = 0.923	**16.15** **(3.47, 75.23)** **p < 0.001**
Very good	2 (4.1)	11 (24.4)		3 (10.0)	2 (9.5)		
Good	15 (30.6)	10 (22.2)		10 (33.3)	8 (38.1)		
Fair	15 (30.6)	9 (20.0)		11 (36.7)	7 (33.3)		
Poor	11 (22.5)	0		3 (10.0)	1 (4.8)		
Very poor	3 (6.1)	0		1 (3.3)	2 (9.5)		
**Linear Regression**	***marginal mean*** ***(95% CI)***		***β*** ***(95% CI)*** ***p-value***	***marginal mean*** ***(95% CI)***		***β*** ***(95% CI)*** ***p-value***	***β*** ***(95% CI)*** ***p-value***
**Servings of fruits and vegetables**	3.33 (3.02, 3.65)	4.01 (3.68, 4.35)	**0.68** **(0.33, 1.03)** **p < 0.001**	3.12 (2.69, 3.56)	3.21 (2.69, 3.74)	0.09 (−0.43, 0.61) p = 0.743	0.59 (−0.04, 1.21) p = 0.066
**Frequency of healthy food consumption**	2.97 (2.85, 3.10)	3.23 (3.10, 3.36)	**0.26** **(0.12, 0.40)** **p < 0.001**	2.93 (2.75, 3.11)	2.98 (2.77, 3.20)	0.06 (−0.16, 0.27) p = 0.604	0.20 (−0.06, 0.46) p = 0.126
**Barriers to healthy eating**	2.68 (2.41, 2.95)	2.12 (1.84, 2.41)	**−0.56** **(−0.87, −0.25)** **p < 0.001**	2.58 (2.20, 2.97)	2.67 (2.21, 3.13)	0.08 (−0.38, 0.55) p = 0.723	**−0.64** **(−1.21, −0.08)** **p = 0.03**
**Shopping, cooking and eating Behaviors**	3.29 (3.08, 3.49)	3.67 (3.45, 3.89)	**0.38** **(0.12, 0.65)** **p = 0.005**	3.20 (2.91, 3.50)	3.53 (3.18, 3.87)	0.32 (−0.07, 0.72) p = 0.111	0.06 (−0.41, 0.54) p = 0.798
**Cooking Self-efficacy**	4.20 (4.01, 4.40)	4.66 (4.46, 4.86)	**0.46** **(0.23, 0.68)** **p < 0.001**	4.31 (4.04, 4.58)	4.34 (4.02, 4.66)	0.02 (−0.31, 0.36) p = 0.885	**0.43** **(0.03, 0.84)** **p = 0.037**

Notes: Mixed-effects ordinal logistic and linear regression adjusted for age, sex, and race/ethnicity.

**Table 3:** Multivariate adjusted mixed-effects linear regression assessing the association of intervention on anthropometric and clinical outcomes.

	Intervention					Control					
	Baseline		Post-Intervention		Within Group Changes	Baseline		Post-Intervention		Within Group Changes	Between Group Changes
Model Estimates	*n*	*marginal mean* *(95% C.I.)*	*n*	*marginal mean*	*β (95% C.I.)* *p-value*	*n*	*marginal mean*	*n*	*marginal mean*	*β (95% C.I.)* *p-value*	*β (95% C.I.)* *p-value*
**BMI**	45	40.02 (36.85,43.19)	40	39.67 (36.50, 42.84)	−0.35 (−0.73, 0.04) p = 0.076	28	36.45 (32.00, 40.91)	26	36.26 (31.80, 40.72)	−0.20 (−0.72, 0.33) p = 0.465	−0.15 (−0.80, 0.50) p = 0.643
**HbA1c**	40	8.40 (7.63, 9.17)	33	7.60 (6.79, 8.41)	**−0.80 (−1.51, −0.09)** **p = 0.027**	24	8.80 (7.69, 9.91)	14	8.25 (7.00, 9.50)	−0.55 (−1.61, 0.51) p = 0.308	−0.25 (−1.52, 1.02) p = 0.701
**SBP**	44	130.82 (125.09, 136.54)	39	124.93 (118.88, 130.98)	−5.89 (−13.33, 1.56) p = 0.121	27	128.33 (120.26, 136.40)	25	132.09 (123.69, 140.50)	3.76 (−6.44, 13.97) p = 0.470	−9.65 (−22.29, 2.98) p = 0.134
**DBP**	44	77.80 (74.94, 80.66)	39	75.23 (72.21, 78.25)	−2.57 (−6.25, 1.10) p = 0.170	27	78.68 (74.65, 82.71)	25	79.90 (75.70, 84.10)	1.22 (−3.81, 6.26) p = 0.634	−3.79 (−10.03, 2.44) p = 0.233

Notes: Mixed-effects linear regression adjusted for age, sex, and race/ethnicity. Changes in medication were tested, but did not change estimates by more than 10%. Sample sizes represent available data in the electronic medical record during a 90-day window at each collection time point; lack of available biometric data did not exclude individuals from the study.

### Program enrollment

Qualitative interviews with program participants (n = 14) revealed several motivating factors for enrolling in the culinary classes. The primary motivators were a desire to change their diet and a health benefit expectation. Participants also noted that they wanted to learn different cooking methods, especially those geared toward diabetes patients, and were excited about the online format of the classes, which minimized driving and allowed family members to participate.

### Facilitators and barriers to class participation

Participants reported that text message reminders helped them attend and prepare for classes, as they may have otherwise forgotten. Texting generally made it easy to communicate with program staff and reference class information at their convenience.

The virtual format of the classes reduced barriers related to transportation and travel time, which made the program more convenient, and the ability to cook in their kitchens ensured their comfort. One participant described the convenience of the virtual format, stating: *“I think I would have had a hard time getting to the class on time. You never know how traffic can be. One day you can get there on time, and another day you can be stuck in traffic for about an hour or something.”* Some participants experienced difficulties with the technology, including joining the virtual meeting, faulty internet connections, and issues with the sound. We provided technical assistance at every session, which participants reported was helpful and facilitated their participation. Additionally, the assistance with technology further encouraged their participation in the program more generally, as one participant expressed: *“it was the interest that was shown to me, about me, really made me excited, because it feels good when someone wants to offer you help, especially when you need it.”*

### Shopping for ingredients

The gift cards were noted as essential to buy the food needed to participate, especially as most participants (84% of the sample) were food insecure. One participant said, *“my budget is really tight, and so the amount that was given was enough and it helped out a lot.”* A few participants reported that the gift cards gave them more options, as they otherwise would have been limited to buying the lowest-cost options. Many participants described enjoying the in-person grocery shopping experience and learning to navigate the store to locate unfamiliar ingredients. Other participants managed grocery shopping by ordering online and having their groceries delivered.

### Learning facilitators

Participants emphasized that they found the hands-on and visual components of the classes most helpful, with watching and practicing nutritious meal preparation helping them build skills. One participant stated, “*I like enjoying and having that involvement in the class… us all participating and doing something instead of just reading… The experience that I got from the cooking class, I learned a whole lot more, and I was able to incorporate that more into my daily life and how I cook and I feed me and my family now.”* Participants reported that learning alongside others improved their experience, as they benefited from hearing peers’ experiences and the instructor’s responses to group questions. Sharing the results of their meal preparation and engaging in interactive discussions with their peers was commonly cited as a class component that participants enjoyed and found helpful. At least half of the participants expressed that the instructor significantly impacted the class experience. They described the instructor as patient, engaging with the participants in discussions, knowledgeable, and easy to understand.

### Program recipes

Several participants expressed positive feedback about the recipes, noting that they enjoyed the combination of familiar and new foods, that their families found the meals appealing, and that they appreciated the quick and simple preparation methods. They also appreciated the flexibility and guidance in modifying recipes, which allowed them to tailor meals to individual tastes, ingredient availability, or cultural preferences, while still encouraging them to try new foods and cooking techniques. Some participants highlighted that a few recipes included foods they or their families were unfamiliar with or disliked.

### Changes in perceptions

Participants reported previously believing or experiencing that healthier food options were more expensive than less healthy alternatives. Participation in the program helped some individuals realize that healthy eating was more affordable and healthy cooking took less time than they had anticipated. One participant described this as follows:

*“Prior to I really had absolutely no confidence because I always assumed that eating healthier would equate to having to spend more money and it would be harder, it would be something harder to prepare and take a long time to prepare. Whereas with the class I learned that it doesn’t have to be very expensive. And it’s not as time consuming as I would have thought.”*

Nonetheless, for others, budgetary realities remained a significant barrier, as illustrated by one participant: *“I could take $40 and go get the ingredients and prepare that meal for that night for the class. But that’s just for one night. What am I going to do for the next 2 or 3 nights? So if I got $40, I’m gonna go in and try to make it stretch.“*

Others reported a belief prior to participating in the program that cooking healthy food would be difficult and would not taste good, with one person saying: *“changing the way you approach food and what you eat was the daunting part. Changing my whole attitude about food… But also not knowing if we would like the taste or not, I think is a thing that everyone had to look at, you know, thinking, oh, I don’t know if I’m going to like this, but the recipes were quite pleasing, so it turned out well.”*

## Discussion

This study evaluated the feasibility, acceptability, and preliminary impact of a culturally inclusive, virtual culinary medicine intervention—NCCM—on psychosocial, behavioral, and biometric outcomes among adults living with type 2 diabetes and elevated BMI. The findings demonstrate that a brief, five-week virtual program was feasible and acceptable, and moreover, can significantly improve perceived health, cooking self-efficacy, and reduce perceived barriers to healthy eating, even after adjusting for demographic differences between intervention and control groups.

These results align with prior research indicating that culinary medicine interventions can enhance nutrition-related knowledge, skills, and confidence, particularly when delivered in an engaging, hands-on format [30–32]. The NCCM program’s emphasis on culturally relevant recipes and financial support for ingredients likely contributed to its acceptability and effectiveness. Notably, the virtual delivery model increased accessibility and convenience, especially for participants facing transportation or mobility challenges—an important consideration for scaling interventions in underserved communities and in large metropolitan areas with traffic. However, challenges such as continued technological barriers despite technical assistance highlight areas for refinement in future iterations of the program.

Although improvements in fruit and vegetable intake and healthy food behaviors did not reach statistical significance in adjusted models, the consistent directionality of effects suggests meaningful behavioral shifts. These trends, coupled with qualitative feedback, underscore the potential of culinary medicine to influence dietary habits [33,34]. Participants reported increased confidence in preparing healthy meals, greater willingness to try new foods, and a shift in perceptions around the cost and complexity of healthy eating—indicating that several commonly cited barriers to healthy eating were overcome [35–37].

Biometric outcomes, including HbA1c, also trended in the expected direction, with a significant reduction in HbA1c observed in the intervention group from pre- to post-test. While changes in biometric variables were not statistically significant when comparing the intervention to the control group, the short duration of the follow-up period may have limited the ability to detect changes in these clinical markers. Future studies with longer follow-up and larger sample sizes are warranted to assess sustained impacts on metabolic health, especially as the landscape for “Food is Medicine” continues to grow [38].

This study has several limitations. First, the quasi-experimental design and non-randomized assignment introduce the potential for selection bias and confounding. Although we adjusted for key demographic differences, unmeasured variables may have influenced outcomes. Second, we conducted complete-case analyses, limiting the sample due to loss to follow-up. We conducted additional analyses (not shown) between those who were retained and those who were lost to follow-up and found no statistically significant differences in demographic variables at baseline. Third, the relatively small sample may have limited statistical power to detect changes and limited our ability to examine how intervention dose and fidelity impacted outcomes. The sample was further limited for biometric outcomes, where data were not available in the EMR. The use of usual care clinical data (as opposed to paying for blood work) enabled this pilot study to be affordable and well-integrated into the clinic system, but limited the completeness of the data. We extracted medication data from the EMR over a one-year period, which allowed us to assess prescribed medications at baseline and post-intervention. However, using EMR data does not provide a complete picture of which medications were filled and taken as prescribed. Despite limitations, we assessed medication and changes in medication usage, testing the latter as a covariate in the biometric models. However, the addition of changes in medication usage did not affect estimates by more than 10%, and the variable was ultimately removed from the final model. Future research could pair EMR data with extensive patient-reported medication history at each assessment. Despite these limitations, the integration of quantitative and qualitative data strengthens the validity of our findings and provides a nuanced understanding of participant experiences in the program.

## Conclusions

The 5-week virtual culinary medicine intervention was feasible, acceptable, and improved important psychosocial and behavioral variables related to healthy cooking and eating in a diverse patient population. The NCCM intervention demonstrates promise as a scalable, culturally responsive strategy to support dietary behavior change among patients with type 2 diabetes and elevated BMI.

## Supporting information

S1 Table Crude mixed-effects regression assessing association of intervention with psychosocial and behavioral outcomes. (DOCX)

S2 Table Crude mixed-effects linear regression assessing the association of intervention on anthropometric and clinical outcomes. (DOCX)

S1 File Survey data in excel. (XLS)

S2 File Biometric data in excel. (XLS)

S3 File Study protocol. (PDF)

S4 File CONSORT checklist. (DOCX)
