# Supplementary material for: Impact of a culinary medicine intervention on diet and health metrics in patients with type 2 diabetes and elevated body mass index
Source: PLoS One. 2026 May 20;21(5):e0347040. doi: 10.1371/journal.pone.0347040 (PMC13189329; doi:10.1371/journal.pone.0347040)
Supplement: S1 Table — (DOCX) [file pone.0347040.s001.docx]

Supplemental Table 1. Crude Mixed-Effects Regression Assess Association of Intervention with Psychosocial and Behavioral Outcomes

|  | **Intervention Group** | | | **Comparison Group** | | |  |
| --- | --- | --- | --- | --- | --- | --- | --- |
| **Variable** | **Baseline** | **Post- Intervention** | **Within Group Changes** | **Baseline** | **Post- Intervention** | **Within Group Changes** | **Between Group Changes** |
| **Ordinal Logistic Regression** | ***n (%)*** | | ***Odd Ratio (95% CI) P-value*** | ***n (%)*** | | ***Odd Ratio (95% CI) P-value*** | ***Odd Ratio (95% CI) P-value*** |
| **Perceived Health** |  |  |  |  |  |  |  |
| Excellent | 3 (6.1) | 15 (33.3) | 14.99 (5.84, 38.49) **p< 0.001** | 2 (6.7) | 1 (4.8) | 0.94 (0.33, 2.73) p=0.914 | 15.89 (3.83, 65.89) **p< 0.001** |
| Very good | 2 (4.1) | 11 (24.4) |  | 3 (10.0) | 2 (9.5) |  |  |
| Good | 15 (30.6) | 10 (22.2) |  | 10 (33.3) | 8 (38.1) |  |  |
| Fair | 15 (30.6) | 9 (20.0) |  | 11 (36.7) | 7 (33.3) |  |  |
| Poor | 11 (22.5) | 0 |  | 3 (10.0) | 1 (4.8) |  |  |
| Very poor | 3 (6.1) | 0 |  | 1 (3.3) | 2 (9.5) |  |  |
| **Linear Regression** | ***marginal mean (95% CI)*** | | ***β (95% CI)  p-value*** | ***marginal mean (95% CI)*** | | ***β (95% CI) p-value*** | ***β (95% CI) p-value*** |
| **Servings of fruits and vegetables** | 3.31 (3.00, 3.61) | 3.94  (3.61, 4.26) | 0.63 (0.27, 1.00) **p=0.001** | 3.31  (2.92, 3.69) | 3.12  (2.66, 3.58) | -0.19 (-0.68, 0.31) p=0.459 | 0.82 (0.20, 1.434) **p=0.009** |
| **Frequency of healthy food consumption** | 2.96  (2.85, 3.08) | 3.20 (3.08, 3.33) | 0.24 (0.10, 0.38) **p=0.001** | 3.01 (2.86, 3.16) | 2.99  (2.81, 3.17) | -0.01 (-0.21, 0.18) p=0.886 | 0.25 (0.01, 0.50) **p=0.039** |
| **Perceived barriers to healthy eating** | 2.72 (2.46, 2.97) | 2.24 (1.97, 2.51) | -0.48 (-0.78, -0.17) **p=0.002** | 2.43  (2.10, 2.77) | 2.55 (2.17, 2.94) | 0.12 (-0.31, 0.55) p=0.578 | -0.60 (-1.13, -0.07) **p=0.027** |
| **Shopping, cooking and eating Behaviors** | 3.30 (3.09, 3.51) | 3.64 (3.42, 3.87) | 0.35 (0.06, 0.63) **p=0.017** | 3.16  (2.88, 3.44) | 3.39 (3.07, 3.71) | 0.23 (-0.16, 0.62) p=0.249 | 0.12 (-0.37, 0.60) p=0.637 |
| **Cooking Self-efficacy** | 4.21 (4.01, 4.41) | 4.68  (4.47, 4.89) | 0.47 (0.24, 0.70) **p<0.001** | 4.22  (3.96, 4.47) | 4.17 (3.88, 4.46) | -0.04 (-0.37, 0.28) p=0.790 | 0.51 (0.11, 0.92) **p=0.012** |
