# Supplementary material for: Impact of a culinary medicine intervention on diet and health metrics in patients with type 2 diabetes and elevated body mass index
Source: PLoS One. 2026 May 20;21(5):e0347040. doi: 10.1371/journal.pone.0347040 (PMC13189329; doi:10.1371/journal.pone.0347040)
Supplement: S2 Table — (DOCX) [file pone.0347040.s002.docx]

Supplemental Table 2. Crude Mixed-Effects Linear Regression Assessing the association of intervention on anthropometric and clinical outcomes

|  | **Intervention** | | | | | **Control** | | | | |  |
| --- | --- | --- | --- | --- | --- | --- | --- | --- | --- | --- | --- |
|  | **Baseline** | | **Post-Intervention** | | **Within Group Changes** | **Baseline** | | **Post-Intervention** | | **Within Group Changes** | **Between Group Changes** |
| **Model Estimates** | *n* | *marginal mean* | *n* | *marginal mean* | *β (95% C.I.) p-value* | *n* | *marginal mean* | *n* | *marginal mean* | *β (95% C.I.) p-value* | *β (95% C.I.) p-value* |
| **BMI** | 45 | 39.73 (36.85, 42.61) | 40 | 39.38 (36.49, 42.26) | -0.35 (-0.71, 0.01) p=0.058 | 28 | 35.58 (31.95, 39.21) | 26 | 35.38 (31.75, 39.01) | -0.20 (-0.65, 0.25) p=0.382 | -0.15 (-0.73, 0.43) p=0.615 |
| **HbA1c** | 40 | 8.52 (7.82, 9.22) | 33 | 7.75 (7.01, 8.48) | -0.78 (-1.44, -0.11) **p=0.022** | 24 | 8.34 (7.43, 9.26) | 14 | 7.86 (6.77, 8.94) | -0.49 (-1.47, 0.49) p=0.330 | -0.29 (-1.48, 0.90) p=0.632 |
| **SBP** | 44 | 130.66 (125.18, 136.14) | 39 | 125.15 (119.35, 130.95) | -5.51 (-12.52, 1.50) p=0.124 | 27 | 128.39 (121.40, 135.38) | 25 | 133.55 (126.30, 140.81) | 5.16 (-3.68, 14.00) p=0.253 | -10.67 (-21.96, 0.61) p=0.064 |
| **DBP** | 44 | 78.86 (76.09, 81.63) | 39 | 76.14 (73.22, 79.07) | -2.72 (-6.14, 0.70) p=0.119 | 27 | 76.79 (73.26, 80.32) | 25 | 78.89 (75.24, 82.55) | 2.11 (-2.20, 6.42) p=0.338 | -4.82 (-10.32, 0.67) p=0.086 |
