# Supplementary material for: Impact of a culinary medicine intervention on diet and health metrics in patients with type 2 diabetes and elevated body mass index
Source: PLoS One. 2026 May 20;21(5):e0347040. doi: 10.1371/journal.pone.0347040 (PMC13189329; doi:10.1371/journal.pone.0347040)
Supplement: S3 File — (PDF) [file pone.0347040.s005.pdf]

**Protocol Title:**	Nourishing the Community Through Culinary Medicine- Acres Homes
**Principal Investigator:**	Natalia Heredia, PhD, MPH, Assistant Professor, Department of Health Promotion and Behavioral Sciences
**Study Coordinator:**	Sarah Bentley, MPH
**Population:**	132 adult patients (18-75 years) with type 2 diabetes and elevated body mass index receiving care from UT Physicians clinics
**Number of Sites:**	Various UT Physicians clinics- single system
**Study Duration:**	October 2023 to September 2024
**Subject Duration:**	12 months participation with approximately 3 months of active participation and the remainder of time for passive follow-up on patient outcomes via electronic medical records.

General Information

- Nourishing the Community Through Culinary Medicine Study is a pilot study to implement a virtual culinary medicine intervention to low income, ethnically diverse adults with diabetes and elevated body mass index. A pre-post survey design and clinical electronic medical record data (HbA1c, Body Mass Index, Blood Pressure, HDL, LDL and Triglycerides) will be used to evaluate patient outcomes.

Background Information

- Over 34 million (13%) adults in the United States (US) have diabetes mellitus (DM), with rates climbing over 26% among adults aged 65 years or older and disproportionately higher rates among many Texas residents. (CDC, 2020; Schuler & Koka, 2019). The disparity grows stark when broken down by age among Texas residents, with 42% and 53.9% of adults aged 45 or older suffering from DM in the Dallas and Houston regions respectively (BRFSS, 2018). Further, adults with DM carry a 50% higher risk of mortality, have more health complications, and spend more than $9,500 yearly on medical costs than their counterparts. (Rowley, 2017). In addition, the CDC estimates more than 34% of adults are currently afflicted with pre-diabetes and at risk of developing DM. Alarmingly, the prevalence of DM is forecast to increase by more than 50% over the next decade (Rowley, 2017).
- Although consuming a healthy diet is a well-supported and low-cost approach for reducing the risk of Type 2 DM (Hu et al., 2020), less than 10% of adults in the U.S. actually follow this approach (USDA 2015; Wilson et al., 2016). Low levels of food literacy coupled with poor dietary consumption behaviors among the US population warrant the need for innovation in nutrition education and intervention approaches (Downer et al., 2020; Etherton et al., 2014; Van Horn et al., 2019). Culinary Medicine (CM) is an emerging educational and nutritional approach that adds to current nutrition education and interventions by incorporating both, the practical hands-on preparation skills and pleasure of food, and the scientific knowledge of how nutrition and dietary patterns affect health outcomes (Birkhead, et al., 2014; La Puma et al., 2016; Polak et al., 2016; Barkoukis et al., 2019).

UTHealth Houston
IRB NUMBER: HSC-SPH-23-0810
IRB APPROVAL DATE: 04/08/2024

- There is currently a critical gap of evidence-based "how-to" nutrition information that is tailored for culturally diverse, low-income minority populations across Texas. Furthermore, lifestyle interventions are needed to mitigate chronic disease such as type 2 DM among low-income minority populations, who are at a disproportionately higher risk for these conditions. This can be done by strengthening clinic-community linkages to offer lifestyle-based prevention services for their patients. We will build upon existing resources at UTHealth Nourish program (McWhorter et al., 2019) which implements interdisciplinary evidence-based CM curricula across sectors in Harris County.

Objectives

- The purpose of this study is to implement a five-session hands-on virtual CM program (to be held weekly or bi-weekly) among patients with diabetes and elevated body mass index receiving care UT Physicians clinics serving the Acres Homes neighborhood, and comparing their outcomes to patients in usual care from UT Physicians clinics outside of the Acres Homes service area.
- The overall goals of this project are to:
    - To implement adapted virtual CM curriculum among target population
    - To assess if program participation improves participant dietary behaviors, nutrition knowledge, and cooking skills and behaviors above standard of care.
    - To assess if program participation improves patient levels of HbA1c, Body Mass Index, Blood Pressure, HDL, LDL and Triglycerides above standard of care.
    - To assess if social determinants of health modify the association between participating in the program and any of the aforementioned outcomes.
    - To determine the feasibility and reproducibility of virtual synchronous CM classes.

Study Design

- This study is a pilot study with a quasi-experimental design using assigned intervention and control clinics.
- Study participants will be evaluated prior to and immediately after the program via pre and post survey.
- EMR data will be reviewed at baseline and completion of program
- This one-year project will include:
    a) conducting the virtual CM program for approximately 66 intervention patients with diabetes receiving care at UT Physician Clinics serving the Acres Homes neighborhood in Houston
    b) conducting program evaluation (pre- and post-test surveys and EMR data) using data from those 66 intervention participants and 66 control participants recruited from UT Physicians clinics outside of the Acres Homes service area
- The pre-post survey will evaluate participants' dietary behaviors, nutrition knowledge, cooking skills and behaviors along with demographic information. The EMR data will evaluate improvements in clinical data: HbA1c, Body Mass Index, Blood Pressure, HDL, LDL and Triglycerides. The results will be used to determine if virtual CM education can be effective in improving certain health outcomes, dietary behaviors and cooking-related constructs.
- There are a few minimal risks known to taking part in this study. Knives will be utilized raising the risk for minor cuts. Stove tops, ovens, microwaves, and other kitchen equipment will be utilized raising the risk for minor burns. Food items containing known allergens will be utilized raising the risk for allergic reaction. We anticipate participation in this study to be of minimal risk and do not expect our program or surveys to cause emotional distress.

UTHealth Houston
IRB NUMBER: HSC-SPH-23-0810
IRB APPROVAL DATE: 04/08/2024

Study Population

- It is anticipated that program participants will be low-resource and ethnically diverse, reflective of UT Physicians patients at the clinics serving the Acres Homes neighborhood of Houston.
- Inclusion Criteria:
    - Receiving care at UT Physicians clinics;
    - Adult patients (18 to 75 years) diagnosed with type 2 diabetes and elevated body mass index (>=25);
    - HbA1c labs and clinic-assessed weight completed within the last 3 months;
    - English speaking or Spanish speaking.
    - Can obtain groceries before each class (intervention group only)
- Exclusion Criteria:
    - Patients without the technological support needed to participate (e.g., reliable internet and device - cell phone, tablet or laptop);
    - Patients with an uncontrolled impairment that interferes with ability to participate.
- Recruitment: Patients meeting the aforementioned inclusion criteria will be invited to participate. We will conduct separate recruitment for intervention and control participants- with the intervention participants recruited from the Victory and Greens UT Physicians clinics that serve Acres Homes, and control participants recruited from the Jensen UT Physicians clinic. Jensen has a similar patient population to Victory and Greens, and is relatively close geographically, while still sitting outside of the target neighborhood of Acres Homes. UT Physicians' care coordinators will assess patient eligibility to participate in the program and share either a flyer or an interest form link with eligible patients. Patients will complete the interest form giving UTHealth their contact information. UTHealth staff will reach out to patients and explain the program (time commitment, class days/time). If patient is interested, UTHealth study staff will perform a technology screening since all classes will be held virtually. After screening, UTHealth will send an online informed consent and pre-survey.

Study Procedures

- Program Adaptation and Implementation:
- For this project, we will use the Nourishing the Community through Culinary Medicine curriculum that is available on our Nourish website: https://sph.uth.edu/research/centers/dell/nourish/research-resources/toolkit
- The table below outlines the topics and objectives for the 5 NCCM sessions:

Session	Cooking Technique	Topics and Materials	Learning Objectives	SCT Change Objectives
1	Roasting vegetables and steaming grains.	Handouts: Healthy Eating; Goal setting.<br><br>Videos: My Plate is Your Plate; Small Changes for Health.	• Use safe and effective knife skills to cut a variety of vegetables.<br>• Discuss challenges with managing diabetes.<br>• Identify a healthy plate as ½ fruits and vegetables, ¼ lean protein, and ¼ whole grains.<br>• Understand how to set realistic goals.<br>• Learn how to prepare a balanced family meal.	

UTHealth Houston
IRB NUMBER: HSC-SPH-23-0810
IRB APPROVAL DATE: 04/08/2024

2	Cooking grains pilaf-style and baking.	Handouts: Diabetes management; Carbohydrate counting; Label reading.<br><br>Videos: All About Carbohydrates; Low Blood Glucose; High Blood Glucose.	• Use safe and effective knife skills to cut a variety of vegetables.<br>• Describe what foods and beverages contain carbohydrates.<br>• Learn how to control blood sugar levels using a MyPlate approach.<br>• Learn how to prepare a balanced family meal.	• Outcome expectations of the taste of healthy foods.<br>• Knowledge of healthy eating.<br>• Self-efficacy of preparing healthy foods.<br>• Perceived social support for healthy eating.<br>• Normative beliefs of healthy food.
3	Soups, stews, and preparing dressings.	Handouts: Healthy habits for the family<br><br>Videos: A Family Affair<br>Why Beverages Matter.	• Use safe and effective knife skills to cut a variety of vegetables.<br>• Practice strategies to develop healthy and mindful habits with family.<br>• Learn how to prepare a balanced family meal.	
4	Sautéing, stir frying, and steaming grains.	Handouts: Planning Healthy Meals<br><br>Videos: How to Stock a Healthy Pantry; How to Read Nutrition Facts Labels.	• Use safe and effective knife skills to cut a variety of vegetables.<br>• Practice strategies meal planning and grocery shopping.<br>• Practice reading food labels to encourage better food purchases.<br>• Prepare a balanced family meal.	
5	Sautéing, stir frying, and microwave cooking.	Handouts: Mindful eating.<br><br>Videos: Beyond the Scale<br>Mindful Eating Techniques.	• Use safe and effective knife skills to cut a variety of vegetables.<br>• Practice strategies to develop healthy and mindful habits to control blood sugar.<br>• Describe plans to focus on health versus weight.<br>• Prepare a balanced family meal.	

- To conduct the program virtually, we will do the following
  - First, classes will be streamed live from UTHealth Nourish Program's Teaching Kitchen via Zoom or WebEx platform. Participants will cook and engage virtually (with video and sound on) from their home kitchens via the digital platform.
  - Second, participants will be located across Houston, limiting the ability to provide a "grocery bag". Participants will be provided a $40 grocery card and shopping list per class (5 classes for a total of $200) to purchase ingredients for the cooking sessions. The grocery lists will provide tips and insight to help participants navigate shopping for ingredients.
  - Third, asynchronous virtual educational content (cooking skills videos, animated nutrition education videos, and additional recipes) will be provided to engage and retain participants beyond initial sessions (available at website link).
  - Fourth, to ensure opportunities for participants to engage, classes will be offered during convenient time for participants and recipes will provide enough food for a family of four.
  - Fifth, cohorts will be conducted in either English or Spanish to include a large percentage of clinic patients.
- UTHealth staff will call participant one week prior to classes starting to confirm attendance. If patient confirms attendance, UTHealth staff will send email with class information and gift card e-

UTHealth Houston
IRB NUMBER: HSC-SPH-23-0810
IRB APPROVAL DATE: 04/08/2024

code, grocery list, recipes, etc. UTHealth staff will call patients the day before class and send a text reminder 2 hours prior to the class start time.

– The virtual curriculum will include five 90-minute sessions (to be held weekly or bi-weekly). Participants will also be expected to shop for groceries ahead of the sessions to participate in the program. A gift card will be provided for groceries ($40 per class = $200 total).
– Partnership with UT Physicians sites will allow use of Electronic Medical Record (EMR) data: patient level data for HbA1c, Body Mass Index, Blood Pressure, HDL, LDL and Triglycerides collected as part of their regular standard of care and documented within the EMR. No additional visits, screenings or time will be necessary for participation in the program. Data will be abstracted and shared by clinic site to UTHealth at: baseline (within 90 days of starting the NCCM program) and at program completion (within 90 days of NCCM program completion).
– Informed consent will be obtained electronically via REDcap prior to survey administration. Participants will review the consent documents. Participants will be allowed to contact the Study Coordinator with any questions prior to completing the consent form or the survey. Participants will be allowed to leave the project at any time.
– Participants will be asked to complete an electronic 15-minute survey via REDcap at two timepoints: pre-test survey before the first class and a post-test after the conclusion of the program. The electronic pre- and post-test surveys will collect information on 1) dietary intake, 2) cooking skills, 3) cooking-related psychosocial constructs, 4) diabetes management, and 5) demographics (age, sex, race/ethnicity, education, employment status, preferred language spoken at home, and participation in government assistance programs will be collected at baseline at the start of the program). Survey items are described in detail in the measurement resource table below.

**Primary Outcome- measured at both pre-survey (baseline) and post-intervention (follow-up)**		
HbA1c	Measured by the clinic at baseline and follow-up	
**Secondary Outcomes- measured at both pre-survey (baseline) and post-intervention (follow-up)**		
Other clinical measures	Body Mass Index (height and weight), Blood Pressure, HDL, LDL and Triglycerides measured by the clinic at baseline and follow-up	
Variable (Construct)	Number of items and sample question	Source citation
Perceived Health	1 item<br>E.g. Overall, how would you rate your health in the past four weeks?<br>Response options (1-6) – Excellent to Very Poor	Bowling, A; Just one question: If one question works, why ask several? Journal of Epidemiology & Community Health (2005); 59:342-345.
Fruit & Vegetable consumption	2 item<br>E.g. How many servings of VEGETABLES do you eat or drink each day?<br>Response options (0 to 5): None to 4+ servings per day	Yaroch AL, et al. Evaluation of Three Short Dietary Instruments to Assess Fruit and Vegetable Intake: The National Cancer Institute's Food Attitudes and Behaviors Survey. J Acad Nutr Diet. 2012 Oct;112(10):1570-7.(Link: http://www.ncbi.nlm.nih.gov/pubmed/23017567)
Whole grain consumption	1 item<br>E.g. How many servings of Whole Grains do you eat each day?<br>Response options (0 to 5): None to 4+ servings per day	Adapted from:<br>Yaroch AL, et al. Evaluation of Three Short Dietary Instruments to Assess Fruit and Vegetable Intake: The National Cancer Institute's Food Attitudes and Behaviors Survey. J Acad Nutr Diet. 2012 Oct;112(10):1570-7.(Link: http://www.ncbi.nlm.nih.gov/pubmed/23017567

UTHealth Houston
IRB NUMBER: HSC-SPH-23-0810
IRB APPROVAL DATE: 04/08/2024

Typical food consumption behaviors	7 items:<br>E.g. How often do you typically eat a green salad?<br>Response options (0-4): Not at all to More than once a day	Pinard, Courtney A., et al. "Development and testing of a revised cooking matters for adults survey." American journal of health behavior 39.6 (2015): 866-873.
Perceived Barriers of Eating Fruits and Vegetables	13 items<br>E.g. I don't eat fruits and vegetables as much as I like to because they cost too much<br>Response options (0-4): Strongly agree to strongly disagree	Pinard, Courtney A., et al. "Development and testing of a revised cooking matters for adults survey." American journal of health behavior 39.6 (2015): 866-873.
Eating/cooking/using nutrition labels	10 items<br>E.g. How often do you compare prices before you buy food?<br>Response options (1-5): Never to Always	Pinard, Courtney A., et al. "Development and testing of a revised cooking matters for adults survey." American journal of health behavior 39.6 (2015): 866-873.
Barriers to Healthy Eating	4 items<br>E.g. Cooking healthy food is difficult<br>Response options (1-5): Strongly agree to strongly disagree	Not Previously validated, pilot tested in APHL
Self-efficacy in cooking food and meal planning	5 items<br>E.g. How sure are you that you could use basic cooking techniques (e.g. microwaving, sautéing, roasting).<br>Response options (0-4): Not at all sure to Extremely Sure	Condrasky, M.D., Williams, J.E., Catalano, P.M., & Griffin, S.F. (2011). Development of psychosocial scales for evaluating the impact of a culinary nutrition education program on cooking and healthful eating. Journal of Nutrition Education and Behavior, 43, 511-516.
Diabetes management	16 items<br>E.g. The following statements describe self-care activities related to your diabetes. Thinking about your self-care over the last 8 weeks, please specify the extent to which each statement applies to you.	Schmitt, A., Gahr, A., Hermanns, N., Kulzer, B., Huber, J., & Haak, T. (2013). The Diabetes Self-Management Questionnaire (DSMQ): development and evaluation of an instrument to assess diabetes self-care activities associated with glycaemic control. Health and quality of life outcomes, 11(1), 1-14.
**Tertiary - measured at both pre-survey (baseline) and post-intervention (follow-up)**		
Nutrition knowledge	1 item<br>E.g. When thinking about preparing a plate of food, how much of your plate should be filled with fruits and vegetables?	Not previously validated, pilot tested in APHL

UTHealth Houston
IRB NUMBER: HSC-SPH-23-0810
IRB APPROVAL DATE: 04/08/2024

Covariates- baseline only		
Food Insecurity	2 items<br>e.g., "Within the past 12 months we worried whether our food would run out before we got money to buy more?	Hager, E.R., Quigg, A.M., Black, M.M., Coleman, S.M., Heeren, T., Rose-Jacobs, R., Cook, J.T., De Cuba, S.A.E., Casey, P.H., Chilton, M. and Cutts, D.B., 2010. Development and validity of a 2-item screen to identify families at risk for food insecurity. *Pediatrics*, *126*(1), pp.e26-e32.
Internet Access	4 items<br>e.g., Do you ever go on-line to access the Internet or World Wide Web, or to send and receive e-mail?	From: HINTS 5, Cycle 4 – Section B: Using the Internet to Find Information, in HINTS 5, Cycle 4 (2020) Methodology Report. https://hints.cancer.gov/docs/methodologyreports/HINTS5_Cycle4_MethodologyReport.pdf
Health Literacy	3 items<br>e.g., How often do you have problems learning about your medical condition because of difficulty understanding written information?	Chew, L.D., Bradley, K.A., & Boyko, E.J. (2004). Brief questions to identify patients with inadequate health literacy. Family Medicine, 36(8): 588-594.
Housing insecurity	2 items<br>e.g., What is your living situation today?	From The Accountable Health Communities (AHC) Health-Related Social Needs (HRSN) Screening Tool. Billioux, A., Verlander, K., Anthony, S., & Alley, D. (2017). Standardized screening for health-related social needs in clinical settings: the accountable health communities screening tool. [Discussion paper]. NAM Perspectives. National Academy of Medicine. https://doi.org/10.31478/201705b

- A sub-set of participants from the intervention group (n=15) will be invited to take part in interviews about their experience in the program, including the cooking classes, recipes, recruitment and engagement. We will also try to understand if and what structural barriers they experienced to participating in the study. This will help provide us data as to how the program may need to be tailored.
  - Participants will be offered the opportunity to participate in the interviews while they are still enrolled in the program or immediately after completing the post-survey.
  - They will give consent by texting or emailing us their interest in participating and then scheduling an interview.
  - Participants will receive a $25 gift card for their time.
  - The approximately 30-45-minute interviews will be conducted by trained UTHealth staff and will take place via WebEx.
  - All interviews will be recorded for transcription and subsequent analysis. Spanish interviews will be translated to English by native Spanish speakers. Transcriptions will be coded using standard methods through Dedoose software and deductive thematic analysis will be conducted.
- All patient data will be stored in REDCap and interviews will be saved on the secured share drive.
- Participants will be allowed to contact the principal investigator with any questions. Participants will be allowed to leave the project at any time.

Data and Safety Monitoring

- There are few adverse events expected. Since the classes involve cooking in a participant's home kitchen, there might be injuries with use of knives, other cooking utensils, stoves, and ovens. Participants assume all risk associated with participating in the culinary medicine classes.

UTHealth Houston
IRB NUMBER: HSC-SPH-23-0810
IRB APPROVAL DATE: 04/08/2024

- If adverse events occur, an adverse events form will be completed and submitted to the IRB.

Compensation

- Participants will receive a $25 gift card for enrolling in the study and completing the pre-survey.
- Participants will receive a second $25 gift card for the post-survey.
- Participants will not be compensated for completing the surveys if they received $25 gift cards for completing the surveys in a prior study cohort.
- Participants will receive a $40 grocery card per class (5 classes for a total of $200) to purchase ingredients for the cooking sessions.
- Participants who are repeating a class will not receive a $40 grocery card if they received one for the same class in a prior study cohort.
- Participants who take part in the interviews will receive an additional $25 gift card.
- Participants who are absent for a session will not receive a $40 grocery card for the following session until they confirm with a staff member that they plan to attend the following session.
- Participants who are absent for 2 or more consecutive sessions will not receive a $40 grocery card until they attend another session.

Statistics

- 132 participants are planned to be enrolled, 66 from intervention clinics and 66 from control clinics.
- All participants that enroll in the program will be included in the analysis. Saturation point is considered attendance of 4 out of 5 sessions (80%).
- Statistical analysis will include descriptive and frequency analysis of responses, a pre-post analysis using paired t-test, chi-square test, and Analysis of Covariance to determine changes in biometric and behavioral measures pre to post NCCM program participation.
- Level of significance will be set at $p < 0.05$.
- Qualitative data will be analyzed for themes using standard methods and NVivo software. Responses will be summarized, and a report will be created to provide information to our research investigators and the public health community.

Ethics

- We will obtain informed consent from all interested participants during the technology screening before the first session. The participants will be given a digital consent via REDcap to review and acknowledge, a copy of the consent will be sent to the email provided by the patient. Patients will have the opportunity to pause signing of the consent in order to ask any questions they may have.

Data handling and record keeping

- Electronic Medical Record (EMR) data: Clinic, demographic and healthcare utilization data will be stored in the UT Physicians database. UT Physicians' team members will create custom extract report from the EMR for the participating patients. The report will be sent to UTHealth CMS Certified Data Center as a HL7 using existing UT Physicians protocols. Patient level data for HbA1c, Body Mass Index, Blood Pressure, HDL, LDL and Triglycerides collected as part of their regular standard of care and documented within the EMR will be abstracted by and provided by UT Physicians to the study team at baseline (within 90 days of starting the NCCM program) and at program completion (within 6 months of NCCM program completion).

UTHealth Houston
IRB NUMBER: HSC-SPH-23-0810
IRB APPROVAL DATE: 04/08/2024

- All data will be kept confidential and stored on UTHealth Houston SPH computers on password protected share drives. All survey information will be securely collected and stored in REDCap data management software at UTHealth. Study codes in the form of a unique number will be assigned to each participant to label their response. A separate electronic password protected document that links the study code to subjects' identifying information (name, phone number, email, zip code of residence) will be stored on UTHealth Houston SPH computers on password protected share drives. Only aggregate data will be reported and published.
- Data on EMR outcomes will be linked to survey data based on a series of variables collected (name, DOB, zip code). Once the data is linked, the individual's record will be assigned a dummy ID and all direct identifiers removed for safety issues. A crosswalk that would allow for re-identification will be kept on a separate file stored in an encrypted external drive securely saved under key UTHealth Houston SPH office.
- All interviews will be recorded and transcribed. Transcriptions will be edited to exclude names and other identifying information. All audio files will be destroyed after they are transcribed and analyzed. Audio and transcription files will be stored on UTHealth's secure server, on password protected drives. Data handling procedures will be overseen by Dr. Heredia.

Quality control and assurance

- Respondents' email will be kept on a password-protected computer and can be used to correct ID's if needed.
- The surveys will be administered electronically and all participants will complete the survey prior to and after the intervention program. Any participants that do not attend the last class will be contacted by phone and email to complete the electronic post survey.
  Before analyses are conducted, the raw data will be reviewed for completeness.

Publication Plan

- Investigators at UTHealth will submit manuscripts based on the results of the project to academic journals for publication. All manuscripts will include de-identified data.
- This culinary medicine program will be conducted in partnership with UT Physicians and have the potential to be integrated into existing programs.
- In addition, this program will provide preliminary data that can be used for a full-scale trial.

ATTACHMENTS

1. Study Consent Form
2. Pre/ Post Survey

References:

1. Barkoukis, H., Swain, J., Rogers, C., & Harris, S. R. (2019). Culinary medicine and the registered dietitian nutritionist: Time for a leadership role. Journal of the Academy of Nutrition and Dietetics, 119(10), 1607-1611.

UTHealth Houston
IRB NUMBER: HSC-SPH-23-0810
IRB APPROVAL DATE: 04/08/2024

2. Centers for Disease Control and Prevention. (2020). National diabetes statistics report, 2020. Atlanta, GA: Centers for Disease Control and Prevention, US Department of Health and Human Services,
3. Darmon, N., & Drewnowski, A. (2015). Contribution of food prices and diet cost to socioeconomic disparities in diet quality and health: A systematic review and analysis. Nutrition Reviews, 73(10), 643-660.
4. Hartline-Grafton, H., & Dean, O. (2017). The impact of poverty, food insecurity, and poor nutrition on health and well-being. Washington, DC: Food Research & Action Center,
5. Hu, E. A., Steffen, L. M., Coresh, J., Appel, L. J., & Rebholz, C. M. (2020). Adherence to the healthy eating Index–2015 and other dietary patterns may reduce risk of cardiovascular disease, cardiovascular mortality, and all-cause mortality. The Journal of Nutrition, 150(2), 312-321.
6. McWhorter, J. W., Raber, M., Sharma, S. V., Moore, L. S., & Hoelscher, D. M. (2019). The nourish program: An innovative model for cooking, gardening, and clinical care skill enhancement for dietetics students. Journal of the Academy of Nutrition and Dietetics, 119(2), 199-201.
7. Rowley, W. R., Bezold, C., Arikan, Y., Byrne, E., & Krohe, S. (2017). Diabetes 2030: Insights from yesterday, today, and future trends. Population Health Management, 20(1), 6-12.
8. Schuler, D. A., & Koka, B. R. (2019). Challenges of social sector systemic collaborations: What's cookin in houston's food insecurity space?
9. Sicker, K., Habash, D., Hamilton, L., Nelson, N. G., Robertson-Boyd, L., & Shaikhkhalil, A. K. (2020). Implementing culinary medicine training: Collaboratively learning the way forward. Journal of Nutrition Education and Behavior,
10. Van Horn, L., Lenders, C. M., Pratt, C. A., Beech, B., Carney, P. A., Dietz, W., . . . Kohlmeier, M. (2019). Advancing nutrition education, training, and research for medical students, residents, fellows, attending physicians, and other clinicians: Building competencies and interdisciplinary coordination. Advances in Nutrition, 10(6), 1181-1200.
11. Wilson, M. M., Reedy, J., & Krebs-Smith, S. M. (2016). American diet quality: Where it is, where it is heading, and what it could be. Journal of the Academy of Nutrition and Dietetics, 116(2), 302-310. e1

UTHealth Houston
IRB NUMBER: HSC-SPH-23-0810
IRB APPROVAL DATE: 04/08/2024
